# Nano-based spectroscopic approaches for early diagnosis of Alzheimer’s disease: critical insights into amyloid-β and tau biomarker biology and detection tools

**DOI:** 10.1007/s12551-026-01428-9

**Published:** 2026-03-19

**Authors:** Andra-Sorina Tatar, Alia Colnita, Ioana-Andreea Brezestean, Sanda Boca

**Affiliations:** 1 National Institute for Research and Development of Isotopic and Molecular Technologies, Cluj-Napoca, Romania; 2 Interdisciplinary Research Institute in Bio-Nano-Sciences, Babes-Bolyai University, Cluj-Napoca, Romania

**Keywords:** Alzheimer’s disease, Biomarker, Nanomaterials, SERS, Biosensing

## Abstract

Alzheimer’s disease (AD) is the most prevalent neurodegenerative disorder, and its diagnosis relies primarily on symptoms that emerge long after irreversible neuronal damage. Early detection through reliable quantification of disease-associated protein biomarkers, particularly amyloid-β (Aβ) and tau species, remains a major unmet clinical need. Conventional immunoassays and imaging methods face significant limitations in sensitivity, cost, and suitability for large-scale decentralized screening, thus prompting interest toward nano-enabled spectroscopic biosensing approaches. This review critically examines nano-enabled spectroscopic approaches developed for the detection of Aβ and tau biomarkers, integrating biological context with technological innovation. We compare colloidal nanoparticle-based assays and solid-state nanostructured platforms, with emphasis on surface-enhanced Raman scattering (SERS) and related plasmonic techniques, as well as by the possibility of employing biorecognition strategies. Although many systems achieve ultra-low limits of detection under controlled conditions, we argue that analytical sensitivity alone is insufficient to ensure clinical relevance. Key translational challenges include fabrication reproducibility, batch-to-batch variability, standardization of enhancement metrics, validation in real biological matrices, and the absence of large clinical studies. The increasing incorporation of artificial intelligence for spectral analysis offers opportunities for improved classification, yet introduces concerns regarding dataset size and regulatory validation. By situating nano-spectroscopic biosensing within the biological complexity of early diagnosis of AD biomarkers and the practical constraints of clinical deployment, this review outlines the technological advances achieved to date and the critical steps required for translation: it will depend less on pushing detection limits further and more on achieving reproducibility, manufacturability, cost-effectiveness, and integration into standardized diagnostic workflows.

**Graphical abstract:**

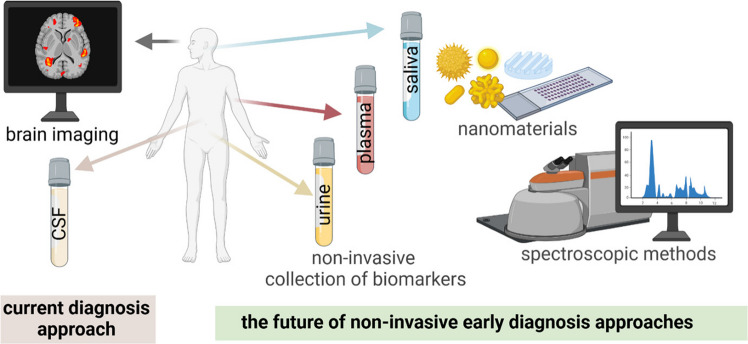

## Introduction

Alzheimer’s disease (AD) is the 7th most common cause of death worldwide and accounts for 60–70% of all dementias. It affects more than 55 million people worldwide, with a predicted doubling every 20 years and a mortality rate over 14 deaths per 1000, in nations such as Bulgaria, Romania, or Latvia. A profound medical and socioeconomic challenge, the total estimated cost of dementias is over 1.3 trillion USD, and is forecasted to reach 2.8 trillion USD in less than 10 years (worldlifeexpectancy.com 2023; WHO 2023).

AD diagnosis generally takes place at the median age of 65 years, in the Early/Mild stage, being characterized by a decline of language skills, perception, movement, and executive functions, and a serious loss of recent memories. Further, in the Middle/Moderate stage, more severe symptoms arise such as the inability to read, write, and properly communicate, to recall older memories and thus to recognize family members, as well as the loss of independence for everyday tasks, together with anosognosia (being unaware of their own diagnosis and inherent limitations). Lastly, in the Late/Severe stage, there is complete dependency on the caregiver as the person is bedridden and unable to feed themselves, due to loss of muscle mass, extremely reduced language, apathy, and exhaustion. Ultimately, infections, pneumonia, cardiac arrest or strokes tend to be the cause of death. Notably, for up to 8 years prior to fulfilling the clinical criteria for an official AD diagnosis, there has been identified another period known as Mild Cognitive Impairment (MCI). This is characterized by subtle symptoms such as a decline in complex and abstract thinking processes, and short-term memory loss, commonly misattributed to stress and normal aging. However, over 90% of amnestic MCI is associated with a later AD diagnosis (Atri 2019). Furthermore, recent work has shown that the inception of AD can actually be recognized up to 20 years before diagnosis (Burnham et al. 2020; Song et al. 2011; Jack et al. 2010), based on molecules that are present in the CerebroSpinal Fluid (CSF), blood/plasma, and other bodily fluids, known as biomarkers, measurable indicators of a biological state or condition (Brazaca et al. 2020; Ghidoni et al. 2018; Agrawal and Biswas 2015).

**Fig. 1:**
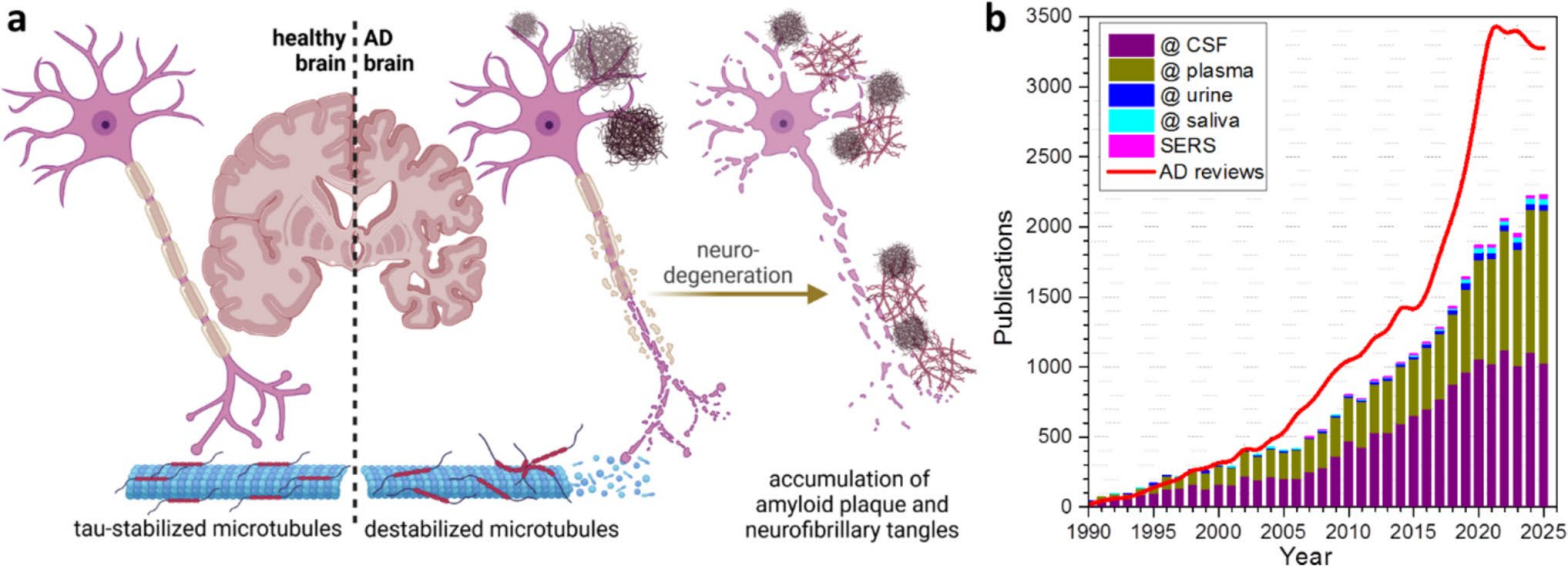
**a** Schematic presentation of AD-specific neurodegeneration. **b** Graphical representation of the number of yearly publications on the subject of AD biomarkers

Even though AD was first described in 1906 (Möller and Graeber 1998), the true complexity of the process by which it develops is yet not fully understood and therefore, despite the numerous research publications on this topic, an accurate and in depth analysis is still required. As generally accepted, the histopathological hallmark of AD is the accumulation of tau-protein based neurofibrillary tangles (NFTs) and amyloid-β peptide (Aβ) based amyloid plaques, resulting in neurodegeneration (Fig. 1a). Currently, conventional detection methods and diagnosis approaches require specialized and expensive imaging techniques such as Magnetic Resonance Imaging (MRI), Computerized Tomography (CT), and Positron Emission Tomography (PET) scans, in combination with unpleasant lumbar punctures for CSF analysis. While Alzheimer’s disease involves a broad spectrum of molecular and cellular biomarkers, this review focuses primarily on amyloid-β and tau proteins, as they remain the most extensively validated pathological hallmarks and the main targets of current nano-spectroscopic sensing strategies. Hence, in Sect. "Alzheimer’s disease (AD) biomarkers" of this review, we deliberately provide an expanded biological overview of Aβ and tau biomarkers, as a deep understanding of their molecular heterogeneity and biofluid dynamics is essential for critically assessing the opportunities and pitfalls of nano-based spectroscopic methods. We discuss their biological activity and clinical relevance when detected in various biological fluids, from CSF, for which the exploration is at a peak (Fig. 1b), to plasma, saliva and urine, as promising prospective sources. Although they represent less invasive approaches, the biomarker concentration in such fluids is greatly reduced, driving the need for nano-enabled spectroscopic approaches, which promise ultra-sensitive detection. The future of biomarker detection and disease diagnosis through the use of modern, faster and cheaper techniques and newly developed assays contrasts the conventional approaches and promises a more easily accessible, time-efficient and cost-effective way. In this regard, Sect. "Nano-based detection tools for AD protein biomarkers" thoroughly reviews the state-of-the-art in nano-based detection methods for protein AD biomarkers, both through colloidal approaches and by the employment of micro and nano detection platforms, a field of growing interest with steadily increasing numbers of publications yearly, as also depicted in Fig. 1b.

Unlike prior reviews focused narrowly on carbon nanomaterials (Rouhi et al. 2023), electrochemical biosensors (Toyos-Rodríguez et al. 2020; Kaushik et al. 2016), or microfluidics (Li et al. 2020), on the detection of amyloid (Kaushik et al. 2016; Vio et al. 2017), or non-Aβ, non-tau biomarkers (Phan et al. 2020), the present work uniquely integrates nanoparticle colloids, nano-fabricated sensing platforms, and spectroscopic modalities into a unified framework, contextualized by the biological complexity and clinical relevance of AD biomarkers. By situating nano-based biosensors within the context of AD key protein biomarkers, this review bridges technological advances in plasmonic enhancement of nano-engineered substrates with the unmet clinical need for sensitive, selective, and minimally invasive diagnostic tools (Nangare and Patil 2022; Gao et al. 2023). While these approaches offer improved limits of detection and enable the use of peripheral fluids such as blood, saliva or urine, their clinical translation remains limited, with only a small number of studies critically addressing the practical challenges of clinical translation regarding biosensor-based early AD diagnosis (Murti et al. 2021; Karki et al. 2021). This work aims to provide both an in-depth biological context for the main protein biomarkers of AD and a critical synthesis of nano-enabled spectroscopic detection strategies, highlighting both the technological opportunities and the translational gaps that must be addressed for meaningful clinical adoption.

## Alzheimer’s disease (AD) biomarkers

Biological markers or *biomarkers* are objectively measurable indicators of the medical state, in contrast to symptoms which are perceived subjectively by the patient. They are measures of biological processes in healthy, pathogenic as well as in therapeutic contexts (Biomarkers Definitions Working Group 2001; Strimbu and Tavel 2010). They range from basic measurements such as pulse and blood-pressure to more complex laboratory blood-work and tissue analyses, all the way to the most cutting-edge state-of-the-art methods such as epigenetics, micro-RNAs, metagenomics, etc. (Wallen et al. 2022; Zotarelli-Filho et al. 2023; Martínez-Iglesias et al. 2021). In 2018, the “A.T.N. diagnosis” system was established, aiming to provide a unified framework for AD diagnosis from a biological perspective as opposed to exclusively clinical symptoms. The acronym stands for Amyloid pathology-Tau pathology-Neurodegeneration, measured by imaging and/or quantification methods (Jack et al. 2016).

In this section, we will focus on AD related protein biomarkers, namely Aβ peptides and tau-proteins, as per the “A.T.N. diagnosis” system, followed by a mention of some other noteworthy and primising examples. We will thoroughly describe their biology and pathology, compare healthy *vs* pathological values, and discuss the advantages and disadvantages of different biological fluids for sampling.

### Protein AD biomarkers

Protein biomarkers represent the molecular core of AD diagnostics, reflecting the underlying neuropathological processes of amyloid aggregation, tau pathology, synaptic dysfunction, and neurodegeneration. While Aβ peptides and tau isoforms are the principal biomarkers incorporated into current diagnostic frameworks and thus detailed within, an expanding panel of protein indicators provides complementary information regarding neuronal injury, astro-glial activation, neuroinflammation, and disease progression, indicating toward the necessity of more multiplexed approaches for the diagnosis of such a complex disease.

#### Amyloid-β peptides

To date, Aβ is the most relevant AD biomarker, in its various sub-types. These peptides are resulted from the degradation of Amyloid Precursor Protein (APP), a transmembrane protein concentrated at synapses, with functions such as neural plasticity (Turner et al. 2003), synapse formation (Priller et al. 2006), and post-injury repair (Plummer et al. 2016). The APP gene is located on chromosome 21, correlating Down syndrome (trisomy 21) with the observed increased rate of AD and Aβ pathology in this patient group (Henson et al. 2020; Sharma 2016; Takata et al. 2008). The APP is naturally cleaved at particular cleavage sites by enzymes called secretases. Thus, the activity of α-, β-, and γ-secretases results in peptide fragments of different sizes, as follows: the native APP is cleaved by α-secretase in the extracellular space close to the cellular membrane, resulting in a membrane-bound fragment known as C83 (83 amino-acid long), and releasing the soluble-APPα fragment. Then, the C83 peptide is cut by the γ-secretase releasing the P3 fragment (33 amino-acid long) and the APP intracellular domain (AICD). This is the non-amyloidogenic APP degradation pathway that predominates in healthy brains. On the other hand, if the activity of the α-secretase is preceded by that of β-secretase, the cleavage site is shifted by 16 amino-acids toward the extracellular region, resulting in a slightly shorter soluble-APP β fragment, and a longer membrane-bound fragment known as C99 (99 amino-acid long). Whence, the γ-secretase releases the AICD, and a 48 or 49 amino-acids fragment. The latter is further processed by γ-secretase (Sanders 2016) through progressive cleavage of groups of three amino-acids, resulting in isoforms of the Aβ peptides which are released into the extracellular, intrasynaptic space: Aβ40 is the most common isoform, characterized by a rather constant concentration in the tissues throughout the stages of life and disease. Aβ42 is the isoform that leads to AD *via* amyloid fibrillogenesis due to its susceptibility to aggregation, oligomerization, and formation of senile plaques (Bachurin et al. 2017; Chen et al. 2017; Fan et al. 2020), as well as a prion-like behavior that perpetuates the pathology (Bredesen 2014). Thus, Aβ42 is considered among the most relevant AD biomarker, along with the Aβ42/Aβ40 ratio for a more consistent tracking of the disease incipience process.

Currently, beside complex and costly imaging approaches, the standard *ex-vivo* method for Aβ detection and quantification with high specificity and sensitivity is based on lumbar punctures and analysis of the CSF using the enzyme-linked immunosorbent assay (ELISA) (Humpel 2011). Also, liquid chromatography mass spectrometry (LC–MS/MS) has also been recently introduced and validated, as a simultaneous assay requiring less steps and using less antibodies compared to ELISA (Seino et al. 2021). Precisely, despite the increased production, the concentration of Aβ42 in the CSF and other body fluids decreases in AD patients, as its` tendency to aggregate into plaques reduces the amount that diffuses outside of the blood–brain-barrier (BBB) (Song et al. 2011). As such, a CSF Aβ42 concentration considered to be indicative of disease would be below 500 pg/ml (Hamley 2012; Van Thanh Nguyen et al. 2021), or in the 25–325 pg/ml range (Mehta et al. 2000), in contrast to healthy controls with Aβ CSF values of 794 ± 20 pg/ml (Sharma 2016; Pérez et al. 2012), or in the 25–1060 pg/ml range, respectively (Mehta et al. 2000).

**Fig. 2:**
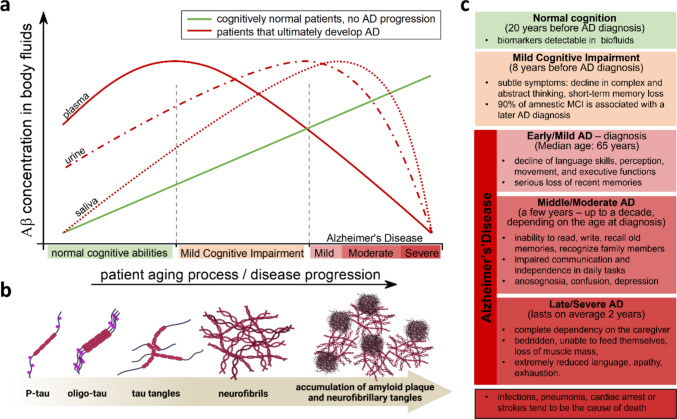
**a** Visual representation of Aβ concentration inverted “u” shape variation throughout the progression of AD, in body fluids such as plasma, urine and saliva (red lines), compared to the linear progression in non-affected individuals (green line). Values are normalized and not to scale. **b** Progressive degradation and aggregation of tau protein concomitant with AD progression, starting from phosphorilation and oligomerisation, resulting in tau tangles and neurofibrils, and ending with massive deposits of NFTs and amyloid plaque. **c** Stages of cognitive decline in the progression of AD, time frames, and distinctive disease features

The decreased CSF Aβ42 levels in AD are correlated with plasma levels decreased below 20 pg/ml according to Pesaresi et al*.* (2006). However, such decreased Aβ levels are not truly relevant for early and pre-AD identification. Importantly though, pre-symptomatic and MCI patients have higher baseline Aβ40 and Aβ42 compared to those that did not ultimately develop AD, with an inverted “u” shape of Aβ42 concentration: increased and increasing values before MCI, that then decrease after the beginning of MCI, and ultimately drop to values below those of cognitively normal people, after AD diagnosis. This is in contrast to the much slower and constant increase of Aβ levels in people unaffected by AD, as shown in a meta-analysis by Song et al*.* (2011), and represented in Fig. 2a. Moreover, it was shown that plasma Aβ42/Aβ40 ratio is more reliable for detecting brain amyloidosis (West et al. 2021; Nakamura et al. 2018), and a better indicative of AD progression than plasma Aβ42 concentration alone, as there is a high variance in quantification methods (Lövheim et al. 2017; Olsson et al. 2016), with individual values tracked throughout life as the most relevant measurement (Song et al. 2011). As such, longitudinal studies have shown plasma Aβ42/Aβ40 abnormalities even before PET scan confirmation of amyloidosis (Burnham et al. 2020; Schindler et al. 2019). Predictions based on a large scale data analysis show that a prescreening blood test for plasma Aβ42/Aβ40 could reduce the number of amyloid PET scans by 59% in cognitively normal patients, reducing the cost and time burden on the medical system (Gao et al. 2023). Through a Class II analysis of 465 participants, in three large AD cohorts (Petersen et al. 2010; Ellis et al. 2009; Janelidze et al. 2016), it was shown that plasma Aβ42/Aβ40 ratio compared to the reference standard based on amyloid PET shows a concordance of 0.84, and that value increases to 0.88 if *APOE ε4* gene status is also taken into consideration. The cutoff values for Aβ42/Aβ40 in these cohorts are 0.123 and 0.125, respectively, with lower values of the ratio being correlated with positive amyloid PET scans (Li et al. 2022). Already applied in the US, the Aβ42/Aβ40 ratio was introduced as a blood based analytically validated method for assessing AD risk, where the ratio cutoff value is considered 0.16 (Quest AD-DetectTM 2023; questdiagnostics.com 2023).

Saliva is promising as a non-invasive biomarker-source biofluid, as it was discussed for sampling for multiple AD biomarkers (Bouftas 2020), and was used for metabolomics analyses that identified novel AD biomarkers (Huan et al. 2018). In a pilot study by Bermejo-Pareja et al*.* (2010), saliva Aβ42 values show an increase from the values of age-sex-matched control patients (2.89 ± 4.96 pg/ml), with more than 2.5 × values in Mild AD patients (7.67 ± 16.25 pg/ml) and 4 × increased values in Moderate AD cases (11.70 ± 34.76 pg/ml), while Aβ40 concentration remains mostly constant (20.82 ± 5.55 pg/ml versus approximately 21.6 pg/ml). Interestingly, for Severe AD cases, the saliva Aβ42 concentration returns to control values (3.03 ± 3.49 pg/ml) in an inverse “u” behavior (Fig. 2a). A meta-analysis of seven studies involving salivary Aβ42 concluded that the molecule may serve as a sensitive biomarker for AD (Fan et al. 2022), but at the same time there are works (Tvarijonaviciute et al. 2020) that contradictory infer a low diagnostic performance despite the decrease of Aβ42 concentration, mainly because the inter-individual variations (i.e. reported standard deviations) often exceed mean values and there is major overlap between diagnosis groups. Thus, assay robustness and reliability in saliva as a diagnostic biofluid should be considered “with a grain of salt” unless clearer cut-off values can be identified, for normalized biomarker concentrations or biomarker ratio values.

As a biofluid that is not regulated by homeostatic mechanisms, urine is theoretically expected to accumulate changes in biomarkers earlier than CSF and blood (Zhang et al. 2018a). Still, less than 1% of the circulating pool of Aβ from circulating plasma is passed daily into the urine (Ghiso et al. 1997). In a study by Takata et al*.* (2008) on the presence of Aβ monomers in the urine of healthy/nonsymptomatic/control, MCI, Mild, Moderate, and Severe AD patients, the rates of Aβ presence follow an inverse “u” behavior, with values of 11%, 62%, 83%, 53%, and 0% respectively. This behavior of this biomarkers’ concentration throughout the disease progression was also observed in plasma and saliva, as discussed herein, and illustrated in Fig. 2a (Song et al. 2011; Bermejo-Pareja et al. 2010).

An important nuance in the relevance of Aβ levels is their non-linear trajectory during AD progression, shown as an inverted “u” across disease stages. In preclinical and prodromal phases, soluble Aβ42 in CSF and peripheral fluids may transiently increase, reflecting enhanced production and impaired clearance before substantial plaque deposition. As disease progresses and amyloid aggregates materialize into insoluble plaques in the brain, soluble Aβ42 levels decline, producing the paradox that advanced AD is associated with low measurable concentrations despite greater total amyloid burden. This non-linear profile complicates biomarker interpretation as absolute Aβ concentrations may be misleading, but at the same time creates a window for early detection and pre-screening of pre-symptomatic AD. Nevertheless, in order to attain clinical patient-specific relevance, the values must be contextualized by disease stage or expressed as ratios (e.g., Aβ42/Aβ40).

#### Tau-proteins

The second most considered AD biomarkers belong to the tubulin associated unit (tau) class. Tau proteins are a group of six isoforms ranging from 352 to 441 amino acids, which emerge through the process of alternative splicing from a gene containing 16 exons, on chromosome 17, known as the microtubule-associated protein tau (MAPT) gene (Buchholz and Zempel 2024). The six isoforms of tau proteins have a ‘projection domain’ toward the N-terminal region which can have different lengths depending on the exons included (both 2 and 3, only exon 2, or neither), located near a proline-rich region toward the center of the protein. The other major functional domain, the ‘microtubule assembly domain’ is contained in the C-terminal region and can have either 4 or 3 repeat domains, depending on the inclusion or exclusion of exon 10 (Wang and Mandelkow 2016; Guo et al. 2017; Šimić et al. 2016). By interacting with tubulins, the monomer proteins that comprise a major component of the cytoskeleton – microtubules, tau proteins modulate the stability of axonal microtubules, and thus the structural and functional integrity of neurites. Pathological modifications of tau proteins, mainly through phosphorylation at more than 40 possible phosphorylation sites (Grundke-Iqbal et al. 1986; Hanger et al. 2009), lead to destabilization of microtubules, additionally sequester normal tau in a prion-like behavior, and result in toxic aberrant tau aggregate structures known as paired-helical-filaments (PHFs) and neurofibrillary tangles (NFTs) (Nichols et al. 2019). The histopathological hallmark of AD is a complex interplay between NFTs and amyloid plaques, as seen in Fig. 2b. Total tau (T-tau) refers to the totality of tau protein present in a measured sample, and phosphorylated tau (P-tau) comprises all P-tau proteins regardless of the particular phosphorylation site, with specific phosphorylated amino-acids marked accordingly, such as P-tau181, P-tau217 or P-tau231 (Hazan et al. 2023; Mattsson-Carlgren et al. 2020; Ashton et al. 2021). Contrary to pathological aggregation, tau is normally a remarkably hydrophilic protein, intrinsically disordered, and natively unfolded, characterized by a low content of transient secondary structures and a high flexibility and mobility. This makes it both noticeably difficult to characterize crystallographically/structurally and eminently complex with regard to its intracellular molecular interactions, a major reason as to why its implication in AD pathology is still not completely understood (Ulamec et al. 2020; Fichou et al. 2019). Nevertheless, T-tau and P-tau are valuable measures of neuronal degeneration, as discussed below.

Currently, the reference method for assessment of tau-based disease progression in AD is PET imaging, but FDA approval of tau PET tracers is lingering (Ossenkoppele et al. 2022). Therefore, we are still in early stages of study before a robust correlation between tau-PET imaging and biofluid AD biomarkers can be made, allowing the switch to non-invasive methods in clinical practice. Beside complex neuroimaging of biomarkers as reference methods, the standard *ex-vivo* method for tau detection and quantification is based on analysis of CSF *via* immunoassay approaches (Annesley 2021). CSF T-tau in AD patients was found to be at approximately 852.3 ± 317.5 pg/ml, in contrast to 372.0 ± 114.2 pg/ml for healthy controls, 402.0 ± 136.1 pg/ml for other non-AD dementia cases and 419.0 ± 240.1 pg/ml for other neuro-degenerative diseases (Kawarabayashi et al. 2020). Other studies also highlight T-tau as a good AD biomarker, with almost doubled values in AD compared to healthy controls (averages of 715 pg/ml *vs* 391 pg/ml) (Puig-Pijoan et al. 2024; Deters et al. 2017). P-tau181 values for healthy control patients have been measured at 47.6 ± 20.3 pg/ml, while AD patients present much higher values of 94.8 ± 55.2 pg/ml. Similarly, P-tau231 varies from 14.7 ± 12.6 pg/ml to 64.4 ± 38.9 pg/ml comparing the CSF of healthy *vs* AD patients (Spiegel et al. 2015). In a study comparing these two subtypes of P-tau, it was concluded that P-tau231 has greater overall specificity and yielded significantly fewer false-positives (Spiegel et al. 2015). Still, the most valuable information seems to come from combinations (ratios) of biomarker concentrations (Fan et al. 2022), such as Aβ42/T-tau or Aβ42/P-tau181, with AD values of 0.81 and 4.76 respectively, in contrast to healthy controls where these ratios were 2.86 and 24.7 respectively (Puig-Pijoan et al. 2024).

Similarly, blood samples contain increased tau protein levels in AD. Using advanced quantification methods, Fossati et al*. *(2019) obtained plasma T-tau values of 3.67 ± 1.06 pg/ml in AD patients and 2.74 ± 0.76 pg/ml in controls (Fossati et al. 2019). Based on a prediction modelling study with data from multiple cohorts, plasma P-tau181 showed gradual increases along the Alzheimer’s disease continuum, from healthy controls to advanced AD cases, measuring 10 ± 3.3 pg/ml and 24.9 ± 7.8 pg/ml respectively. This work also showed this biomarkers’ potential to differentiate AD from other types of dementias and neurodegenerative diseases (Karikari et al. 2020). Other studies also show plasma P-tau181 increasing with the development of AD, with values doubling compared to healthy controls, discussing its effectiveness as a predictor of AD (Hazan et al. 2023; Ashton et al. 2021; Thijssen et al. 2021). Another relevant P-tau biomarker, shown to abruptly increase plasma concentration in patients that later develop the disease, is P-tau217. It is presented as a sensitive marker of the clinical manifestation of AD, capable to predict disease evolution, and differentiate from other neurodegenerative disorders (Mattsson-Carlgren et al. 2020; Thijssen et al. 2021; Telser et al. 2022). Plasma P-tau231 can also distinguish AD cases from other neurodegenerative disorders. It was shown to have an inflection point earlier in disease progression, compared to CSF Aβ and plasma P-tau181, being able to discern between earlier clinical stages of AD neuropathology, and thus identify vulnerable populations below standard threshold for diagnosis (Ashton et al. 2021). Again, the most valuable information regarding prediction of tau pathology and neurodegeneration comes from combinations (ratios) of biomarker concentrations such as T-tau/Aβ42, which was shown by Park et al*.* (2019) to be highly associated with brain volume and metabolism, and highly predictive of brain tau deposition (Park et al. 2019). P-tau217/nonP-tau217 ratio improved plasma biomarker algorithms for screening preclinical individuals for clinical trials (Rissman et al. 2024).

The lack of a uniform protocol for sample collection, conservation, processing and analysis of saliva samples has delayed establishing of a diagnostic panel and thus clinical relevance (Kodintsev et al. 2020). T-tau levels are not always relevant for healthy *vs* AD discrimination, but ratios such as P-tau181/T-tau show significant increase in pathological cases (Lau et al. 2015; Shi et al. 2011). T-tau values were obtained in the 10–20 pg/ml range with just slightly lower values in AD patients’ saliva, while P-tau are an order of magnitude higher, more so in AD cases. Still, there is significant overlap between healthy and AD cases regarding biomarker concentrations and also ratios (Shi et al. 2011), as is the case for Aβ. In a study by Pekeles et al*. *(2018)*,* the authors found P-tau396 to be significantly elevated in AD patients. On the other hand, all P-tau sites and P-tau/T-tau ratios had high variability between patients and generally a low correlation with disease stage based on imagistic methods (Pekeles et al. 2018). These results are corroborated by Aston et al*. *(2018)*,* clearly expressing that salivary T-tau is not suitable as a biomarker due to a large overlap between clinical groups (healthy elderly control, MCI, and AD) with values at 9.6, 9.8 and 12.3 pg/ml respectively.

Studies have proven that tau protein can be renally cleared *via* the kidneys, so a urine-based tau detection diagnostic is potentially possible (Wang et al. 2018). Still, concentrations are significantly lower compared to serum and the studies conducted in this direction are scarce (Chan et al. 2017). In a pilot study by Sun et al*.*, P-tau presence in urinary exosomes has been detected in higher levels compared to healthy controls, at 72.5 pg/ml versus 7.5 pg/ml (Sun et al. 2019). In another interesting paper by Chan et al*.,* T-tau was detected at 107.3 ± 0.15 pg/ml and P-tau181 at 16.22 ± 0.05 pg/ml (Chan et al. 2017). A recent review discusses the potential of wastewater-based epidemiology (WBE) for public health assessment of AD through urine excreted biomarkers such as tau protein, but the low concentration and stability constitute major limitations so far (Armenta-Castro et al. 2024).

Table 1 summarizes the reported concentration ranges of Aβ and tau biomarkers across CSF, plasma, saliva, and urine, along with the practical advantages and limitations of each biofluid for detection. One can readily observe that the literature is far richer for Aβ than for tau, and for CSF compared to the other biofluids, reflecting the historical focus on amyloid pathology and the use of lumbar puncture as the gold-standard diagnostic source. This imbalance underscores where further research efforts are most needed despite their high translational potential, particularly in non-invasive biofluids and non-Aβ biomarkers. As expected, due to the direct contact with the extracellular space of the brain, CSF consistently exhibits the highest analyte concentrations (e.g., Aβ42 in the hundreds of pg/mL and tau in the hundreds to low thousands), enabling robust differentiation between healthy and AD states. Blood levels are dictated by passage through the BBB and subsequent binding or clearance in the periphery, so plasma concentrations are orders of magnitude lower (single-digit to tens of pg/mL ranges), which poses significant analytical challenges despite the appeal of minimally invasive sampling. Additional interference arises from the complexity of the blood matrix, the binding of the biomarkers to the carrier molecules of human serum albumin (HSA) in what is known as the “albuminome” (Kuhlmann et al. 2017), as well as the quick degradation/metabolisation of the proteins of interest (Oyarzún et al. 2021). Salivary and urinary concentrations are further reduced by additional barriers, such as diffusion or transport mechanism, and kidney filtration, respectively. Saliva and urine typically yield extremely low absolute concentrations and high inter-individual variability. The tradeoffs across fluids reflect a key priority for nano-spectroscopic detection: enhance sensitivity to overcome low abundance while ensuring specificity and reproducibility in complex matrices.

**Table 1:** Reported concentration ranges of Aβ and tau in different biofluids, together with the advantages and disadvantages presented by each case

Bio-marker	Bio-fluid	Healthy	AD	Advantages	Disadvantages	Ref
Aβ42	CSF	794 ± 20 pg/mL; 1060 pg/mL	< 500 pg/mL 25–325 pg/mL	strong clinical correlation; established cutoffs	invasive lumbar puncture (LP); limited for large-scale screening	Sharma 2016; Hamley 2012; Van Thanh Nguyen et al. 2021; Mehta et al. 2000; Pérez et al. 2012)
	plasma		< 20 pg/mL 14–51 pg/mL	minimally invasive; scalable	low abundance; high matrix interference; assay variability; strong pre-analytical effects	Song et al. 2011; Pesaresi et al. 2006)
	saliva	2.89 ± 4.96 pg/mL	Early AD 7.67 ± 16.25 pg/mL; Middle AD 11.70 ± 34.76; Late AD 3.03 ± 3.49	non-invasive, patient-friendly collection	low concentrations; high subject variability; nonstandard collection/processing protocols	Bermejo-Pareja et al. 2010)
	urine	Reported as presence rates (pr): 11%	pr: 62% (MCI), 83% (Early), 53% (Middle), 0% (Late)	non-invasive;	very limited excreted fraction (< 1%), poor quantification; few studies	Takata et al. 2008)
Aβ42/Aβ40	plasma	> 0.123–0.16	< 0.123–0.16	minimally invasive; improved specificity; more reliable	quantification of two molecules for calculation of ratio	West et al. 2021; Nakamura et al. 2018; Schindler et al. 2019; Li et al. 2022)
T-tau	CSF	372 ± 114 pg/mL; 391 pg/mL	852.3 ± 317.5 pg/mL 715 pg/mL	well correlated with neuronal damage; established use	invasive sampling; overlap with other neuro-degenerative conditions;	Kawarabayashi et al. 2020; Puig-Pijoan et al. 2024; Deters et al. 2017)
	plasma	2.74 ± 0.76 pg/mL	3.67 ± 1.06 pg/mL	minimally invasive; ultrasensitive assays emerging	extremely low levels; binding to plasma proteins; interference	Fossati et al. 2019)
	saliva	13–17 pg/mL	10–15 pg/mL	non-invasive; easy sampling	overlap across groups; nonstandard protocol; weak correlation with imaging	Shi et al. 2011)
P-tau	saliva	60–75 pg/mL *	75–115 pg/mL *	non-invasive; easy sampling *normalized to total saliva proteins	overlap across groups; nonstandard protocol;	Shi et al. 2011)
	urine	7.5 pg/mL	72.5 pg/mL	non-invasive; 10 × difference in concentration	small pilot study;	Sun et al. 2019)
P-tau181	CSF	47.6 ± 20.3 pg/mL	94.8 ± 55.2 pg/mL	higher specificity for AD pathology vs T-tau; widely used	requires CSF; some inter-assay variability	Spiegel et al. 2015)
	plasma	10 ± 3.3 pg/mL	24.9 ± 7.8 pg/mL	promising blood biomarker with increasing evidence of specificity	low abundance; assay-dependent; needs standardization across platforms	Karikari et al. 2020)
P-tau231	CSF	14.7 ± 12.6 pg/mL	64.4 ± 38.9 pg/mL	greater overall specificity; fewer false-positives	requires CSF; some inter-assay variability	Spiegel et al. 2015)
Aβ42/T-tau	CSF	2.86	0.81	improved specificity; more reliable	invasive LP; limited for large-scale screening	Puig-Pijoan et al. 2024)
Aβ42/P-tau181	CSF	24.7	4.76	improved specificity; more reliable	invasive LP; limited for large-scale screening	Puig-Pijoan et al. 2024)

While Aβ and tau proteins remain the most investigated biomarkers, their concentration dynamics across biofluids demonstrate variability, overlap with other neurodegenerative conditions, and strong dependence on assay platform and pre-analytical handling. Aβ42/Aβ40 ratios provide more robust diagnostic performance than absolute values, but assay-to-assay reproducibility remains poor. Similarly, tau isoforms such as P-tau181 and P-tau217 show promising specificity, yet their stability and relevance in saliva and urine is inconsistent. These challenges underscore why nano-spectroscopic methods, which can enhance sensitivity at very low concentrations, are attractive, but they must also contend with biological variability rather than purely analytical sensitivity.

#### Other relevant AD protein biomarkers

Glial fibrillary acidic protein (GFAP) is an emerging AD protein biomarker (Kim et al. 2023), a cytoskeletal protein overexpressed by reactive astrocytes in pathological conditions, with low plasma levels in healthy people, and steadily increasing values in MCI and AD (Cicognola et al. 2021), well correlated with the decrease in cognitive function. Conversely, salivary GFAP was found significantly decreased in MCI and AD patients in a recent study (Katsipis et al. 2021). Neurofilament Light Chain (NfL) is a marker of general neuronal injury reflecting active neuroaxonal damage. A meta-analysis of 24 papers showed a significant increase of NfL in patients with AD and MCI compared to healthy controls and a clear correlation with cognitive decline (Fan et al. 2023). The utility of NfL as a predictive biomarker for clinical decline with AD was recently confirmed with data compiled from 37 longitudinal statistical models, concluding that higher baseline NfL levels (both plasma and CSF) were consistently associated with greater cognitive decline (Thomas et al. 2025). Alzheimer-associated neuronal thread protein (AD7c-NTP) is a urine-specific protein biomarker first discovered in 1998 (Ghanbari et al. 1998), that is found increased in AD patients compared to age-matched controls (Levy et al. 2007; Zhang et al. 2018b; Lv et al. 2021). More, in a combined computational and experimental study, Yao et al. (2018) identified 3 protein biomarkers expressed in urine of AD patients and involved in AD pathophysiology: osteopontin (SPP1), gelsolin (GSN) and insulin-like growth factor-binding protein 7 (IGTBP7). Acetylcholinesterase is another relevant AD biomarker, as it was found to be significantly increased in the saliva of AD patients compared to healthy controls (Ahmadi‐Motamayel et al. 2019). Recent work by Lv et al*.* (2021) concluded that plasma a-synuclein (a-syn) is significantly increased in AD patients. Sirtuin1 (SIRT1) is a protein involved in neuroprotective pathways and responsible for delaying aging, and was shown as a relevant discriminator between healthy elderly individuals, MCI patients and AD cases with a progressive decrease in plasma concentration along with disease aggravation (Kumar et al. 2013). A more disputed AD protein biomarker is salivary lactoferrin (Lf), an iron-binding protein involved in antimicrobial immune response and inflammation, which decreases in concentration as AD progresses, along with additional extrinsic factors that may influence its concentration (González-Sánchez et al. 2020; Bartolome et al. 2021). However, it was later shown that although Lf is correlated to AD severity, it does not have the capacity to differentiate between other types of dementia (Gleerup et al. 2021). Using a transgenic mouse model, Zhang et al*.* (2018a) identified 29 proteins whose levels differed between wild-type mice and young pre-Aβ plaques APP(swe)/PSEN1^dE9^ mice. In their work, 13 of these proteins have been associated with AD pathogenesis, and 9 have been suggested as novel promising AD biomarkers, all useful for enabling early AD detection, even before the deposition of Aβ plaques. Since neurodegeneration is such a complex biological process, not one biomarker can offer early detection, disease prognosis, and a clear diagnosis. Most biomarkers are non-specifically characteristic to neurodegeneration and neuro-pathologies such as trauma, tumors or strokes, so it is imperative to identify and define co-detection strategies for a clear AD diagnosis.

## Nano-based detection tools for AD protein biomarkers

There is a major societal need for the transfer of the technological advances that ultrasensitive nano-based detection platforms could bring into real-life clinical practice. Capable to compensate for low target concentrations and to achieve detection of extremely low concentrations of analyte, and nanotechnology-based approaches can ensure superior molecular recognition and sufficient amplification levels through plasmonic phenomena such as Surface Enhanced Raman Scattering (SERS), Surface Plasmon Resonance (SPR), or Surface Enhanced Fluorescence (SEF). Plasmons are collective oscillations of conduction electrons in metallic nanostructures that concentrate incident electromagnetic radiation into dramatically enhanced electromagnetic fields. This field confinement enhances light-matter interactions leading to impressive signal amplification in SERS, high sensitivity to refractive index changes in SPR, and modified radiative decay rates that boost SEF, therefore enabling nanosensors to detect biomolecular binding events with sensitivities far beyond those of conventional immunological methods. Below, we present the state-of-the-art nano-based detection tools for AD protein biomarkers, specifically colloidal nanoparticle related assays and nanostructured platforms, respectively. For each, we split the discussion into specific sections on the protein detection, classified into Aβ peptides, tau proteins, and combined Aβ/tau proteins, based on the available literature.

### Colloidal nanoparticle-based assays for AD biomarkers detection

From the class of colloidal metallic nanoparticles, gold nanoparticles (AuNPs) and silver nanoparticles (AgNPs) have been shown to be efficient for a multitude of bio-medical applications, such as bioimaging, diagnostics, drug delivery, repair and regeneration and so on. Biomarker detection falls into a distinct, highly addressed category and this is mostly grace to the optical properties of this class of nanoparticles (Moore et al. 2017; Teleanu et al. 2019; Meenambal and Srinivas Bharath 2020). AuNPs are currently being used as potential tools for the early diagnosis of neurodegenerative diseases, due to their characteristic LSPR which allows the particles to absorb and scatter light with maximum efficiency at certain wavelengths in the visible regime (Kelly et al. 2003). In the particular case of AuNPs one can note their generally accepted biocompatibility which makes them useful for configurations that employ biological entities such as biomarkers without damaging the molecule of interest (Falamas et al. 2025). Combining the unique properties discussed above, AuNPs applicability in bio-sensoristics, including the detection of neurodegenerative disorders or other type of diseases.

#### Amyloid beta (Aβ) peptides

As regarding to the detection of amyloid beta peptides, El-Said et al*.* (2011) developed a SERS assay system based on AuNPs that were electrochemically deposited on an indium tin oxide (ITO) substrate at different heights. With this method, it was possible to determine the concentration of Aβ40 peptide by binding the antibody on the ITO substrate, modified with AuNPs. The best SERS results were obtained for a AuNP matrix height of 91 nm, with a good linear relationship between SERS signal and Aβ concentration. By this method, it was possible to achieve a limit of detection (LoD) of 100 fg/ml.

Xia et al*.* (2019) demonstrated the efficiency of a bifunctional nanoprobe prepared by conjugating AuNPs with Rose Bengal (RB) stain for Aβ42 detection. In this case, the magnitude of the SERS spectral signal of RB molecule which has a particular affinity for Aβ42 could be correlated with the concentration of the peptide of interest. The peptide interaction also induced a notable increase in fluorescence emission. AuNPs functionalized with RB have been used by employing both techniques: SERS-based detection of Aβ42 peptides and fluorescence-based imaging of amyloid plaques.

Jara-Guajardo et al*.* (2020) studied a method for the detection of Aβ through a nanosystem co-administered *in-vivo* with a near-infrared (NIR) fluorescent probe. It used gold nanorods (GNRs) for their SEF effect that was applied for *in-vivo* detection of Aβ. This device could enhance the fluorescence signal of toxic aggregates, allowing improved imaging.

Using membrane-bound protein-coated AgNPs to imitate membrane proteins and the plasmonic characteristics of nanoparticles, Bhowmik et al*.* (2015) investigated Aβ40 aggregation. They reported that the amyloid structure formed a β-turn, which was further encircled by the membrane protein’s β-sheet structure on both sides.

Yang et al*.* (2019) studied AgNPs conjugated with antibodies for Aβ40 and Aβ42, able to detect the biomarkers in a multiplexed manner in human serum, based on SERS. It is feasible to detect concentrations down to 0.25 pg/mL. The detection range of the AgNP nanoprobe-based sandwich assay is two orders of magnitude greater than that of a regular enzyme-linked immunosorbent assay.

For the detection of Aβ biomarkers, chiral plasmonic nanoparticles (ChNPs) have also received exceptional interest. The chiral triangular Au nanorods with a platinum (Pt) framework (l/d-Pt@Au triangular nanorods) were created by Wang et al. (2021). These can be utilized in SERS to identify Aβ monomers and fibrils, with LoD for Aβ42 monomer and fibrils of up to 0.045 _˟_ 10^–12^ M and 4 _˟_ 10^–15^ M, respectively. A chiral d-Pt@Au can also be used to successfully identify Aβ42 in AD patients with an extremely high level of sensitivity (picogram amounts). Thus, (Garcia-Leis and Sanchez-Cortes 2021) showed that LSPR shifts caused by peptide adsorption and self-assembly or by plasmonic hybridization induced by NPs proximity and aggregation, can be readily used to follow the dynamic behavior of an Aβ fragment (25–35) on the surface of NPs. These plasmon-mediated effects showed that the peptide exhibits a typical bimodal adsorption action with two saturation points at 3 and 8 μM.

To investigate the development of AuNPs together with the production of Aβ40 fibrils from monomers in brain tissues, Zhou et al*.* (2020) developed a label-free ratiometric SERS platform. This SERS platform successfully detected Aβ40 monomers and fibrils with high accuracy above concentrations of LoD, which was found at 70 pM and 3 pM, respectively. Another study that demonstrates the quantitative sensitivity of AuNPs towards Aβ40 is presented by Elbassal et al*.* (2017), and investigated interaction kinetics through changes in LSPR. Similar investigations were conducted by Zhang et al*.* at physiological pH (Zhang et al. 2009). They noticed that the protein partially unfolded when it interacted with the AuNPs, which further caused the protein to aggregate as a whole.

In an additional study, using label-free SERS, the detection of AD biomarkers was explored by Demeritte et al*.* (2015). They developed multifunctional graphene oxide-based hybrid nanoplatforms containing nanoparticles coated with a magnetic-plasmonic core. They showed their functionality in detection of Aβ and tau proteins at concentrations up to 100 fg/mL using their SERS "fingerprint". Furthermore, using Fe_3_O_4_@AuMNP and AuNPs, Back et al*.* (Back and Jung 2019) created a SERS-based sandwich immunoassay for rapid and accurate detection of Aβ. In the 1 fM to 1 M region, a linear association between Aβ concentration and SERS signal was found. They obtained a detection threshold substantially lower than 1fM for Aβ.

#### Tau proteins

A possible biomarker for the early detection of Alzheimer’s disease is P-tau396,404, among the earliest phosphorylation event (Ashton et al. 2021). Due to its low abundance, ease of breakdown, and complex formation with other blood proteins or cells, blood P-tau is difficult to detect and is frequently underestimated in traditional plasma-based techniques (Elbassal et al. 2017). Using a highly sensitive and focused SERS-based immunosensor, Zhang et al*.* (2023) were successful in detecting P-tau396,404 in whole blood samples. A reporter with dual colorimetric and Raman signal outputs (4-MBA@AuNP-HRP-R1) was created using a SERS probe and a horseradish peroxidase (HRP)-labeled antibody, resulting in a high signal-to-noise ratio for the immunosensor. In SERS mode, this dual-mode immunosensor was capable to detect blood levels of P-tau396,404 as low as 1.5 pg/mL.

Maurer et al*.* (2020) reported the detection of tau protein by a SERS-based magnetic immunosensor. This was made possible by the creation of the hybrid complex in solutions containing tau protein. The magnetic component of the AuNPs is made up of Fe_x_O_y_ functionalized with polyclonal anti-tau antibody and is capable of separating tau protein from a complex matrix, such as CSF.

#### Multiplexed AD biomarkers

There is a clear lack of nanoparticle-based multiplexed approaches for AD biomarker detection, but one promising modern approach has been developed by Zhang et al*.* (2019). They showed a robust and sensitive multiplexed SERS detection platform for the simultaneous detection of Aβ42 and tau, based on Raman staining of polyA-aptamers connected to AuNPs, with LoDs of 4.2_˟_10^–4^ pM and 3.7_˟_10^–2^ nM for Tau protein and Aβ42 oligomers, respectively.

The colloidal nanoparticle approaches reviewed here exhibit impressive analytical sensitivities, with limits of detection often in the fg/mL to pM range, well below the clinically relevant values, even for plasma or saliva. However, most demonstrations remain confined to spiked samples in buffered media or highly simplified matrices, translation to real biological fluids being underexplored and in need for validation *via* large-scale studies. Moreover, the issues of nonspecific binding, colloidal instability, scalability, and reproducibility across batches are rarely addressed. While AuNPs and AgNPs dominate the field due to their plasmonic properties, reproducibility and biocompatibility considerations must be thoroughly explored for future clinical applications.

### Nanostructured platforms for AD biomarkers detection

In comparison to assays in colloidal suspension, three dimensional (3D) architectures contribute with additional plasmonic enhancement and higher hotspot density favored by the z-axis. A combination of state-of-the-art fabrication techniques such as nano/micro-lithography, colloidal self-assembly, or electrochemistry-based methods can lead to the development of advanced, hierarchical sensing platforms with superior attributes. The advantages include a better signal stability and reproducibility, design flexibility and a wider application range, such as plasmonic micro-laboratories, data storage, security labelling and remote molecular-level airborne sensing (Colniță et al. 2022; Phan-Quang 2019; Phan-Quang 2018; Tatar et al. 2023).

It is noteworthy that the majority of micro/nano detection platform approaches are based on SERS and SPR as the identification and quantification method, whether label-free of tag-based, emphasizing the feasibility of this spectroscopic technique and the power that comes from high levels of enhancement produced by noble-metal plasmonic NPs.

#### Amyloid beta (Aβ) peptides

Indirect SERS-based strategies based on anchored receptors on the surface have been favored in order to overcome the limitations of direct SERS spectroscopy (Porter et al. 2008). One approach by Guerrini et al. (2015) was to exploit the high affinity of these oligomeric species towards specific transition metallic ions (such as Al^3+^ ions) into an engineered SERS sensor. The group fabricated a SERS nanoplatform comprised of self-assembled polystyrene beads decorated with AuNPs functionalized with 4-mercaptobenzoic acid (MBA) and coordinated by Al^3+^ ions which selectively bind Aβ oligomers. The adsorption has been quantitatively correlated by means of SERS measurements.

Yu et al*.* (2018) developed a hybrid-detection platform based on a hexagonal array of Au pyramids covered with a single molecular layer of graphene able to distinguish Aβ40 and Aβ42. The SERS spectra of the two biomarkers in combination with Principal Component Analysis (PCA) facilitated the clear distinction between the two peptide isoforms, revealed the assembly dependent changes in peptide conformation and self-association. Another group also employed a joint use of statistical analysis and SERS mapping in order to make a quantitative assessment of Aβ40 oligomers concentrations in the range 10 nM – 10 μM (Buividas et al. 2015). Using a femtosecond laser, the authors fabricated a 100 × 100 μm^2^ area of self-organized ripple structures coated with a 100 nm Au thin film. It was observed that the SERS intensity signal coming from the Aβ40 oligomers was directly proportional with their concentration.

A sensing platform based on a monolayered Au nanoshells (core radius of ~ 94 nm, with Au shells thickness of ~ 18 nm) film on PVP-modified glass slides and functionalized with sialic film was proposed by Beier et al*.* (2007). The design relied on the high specificity of sialic acid residues for Aβ, while other components from the biological media have been removed. A detection limit in the picomolar range has been attained. In comparison, a detection range of 100 fg/ml to 1 μg/ml was achieved when a SERS-based assay comprising of a AuNPs array of 91 nm in height was electrochemically deposited on ITO substrate and functionalized with an anti-Aβ40 antibody (El-Said et al. 2011).

Another study focused on the SERS differentiation between toxic and non-toxic, synthetic Aβ42 oligomers of different aggregates sizes ranging from 1–2 nm to 5–10 nm in length (Voiciuk et al. 2012). The SERS detection substrate consisted in rough Au electrodes functionalized with self-assembled monolayers (SAMs) of hydrophobic heptanethiol, octadecanethiol and N-(6-mercapto) hexylpyridinium chloride. The study revealed that the smaller Aβ aggregates exhibit a higher effect on the SAMs and overall, on the intensity of the SERS bands, with a marker band at 1387 cm^−1^ assigned to the deprotonated carboxyl groups in Aβ42 oligomers.

Interesting approaches based on amyloid dyes, such as Congo red or Rose Bengal as bio-selective receptors for Aβ detection sensors were summarized by Zhang et al*.* (2020). The conjugation of the dyes with AuNPs or AgNPs in the form of patterned surfaces yields a detection limit of Aβ down to 1 pM (Beier et al. 2007).

**Fig. 3:**
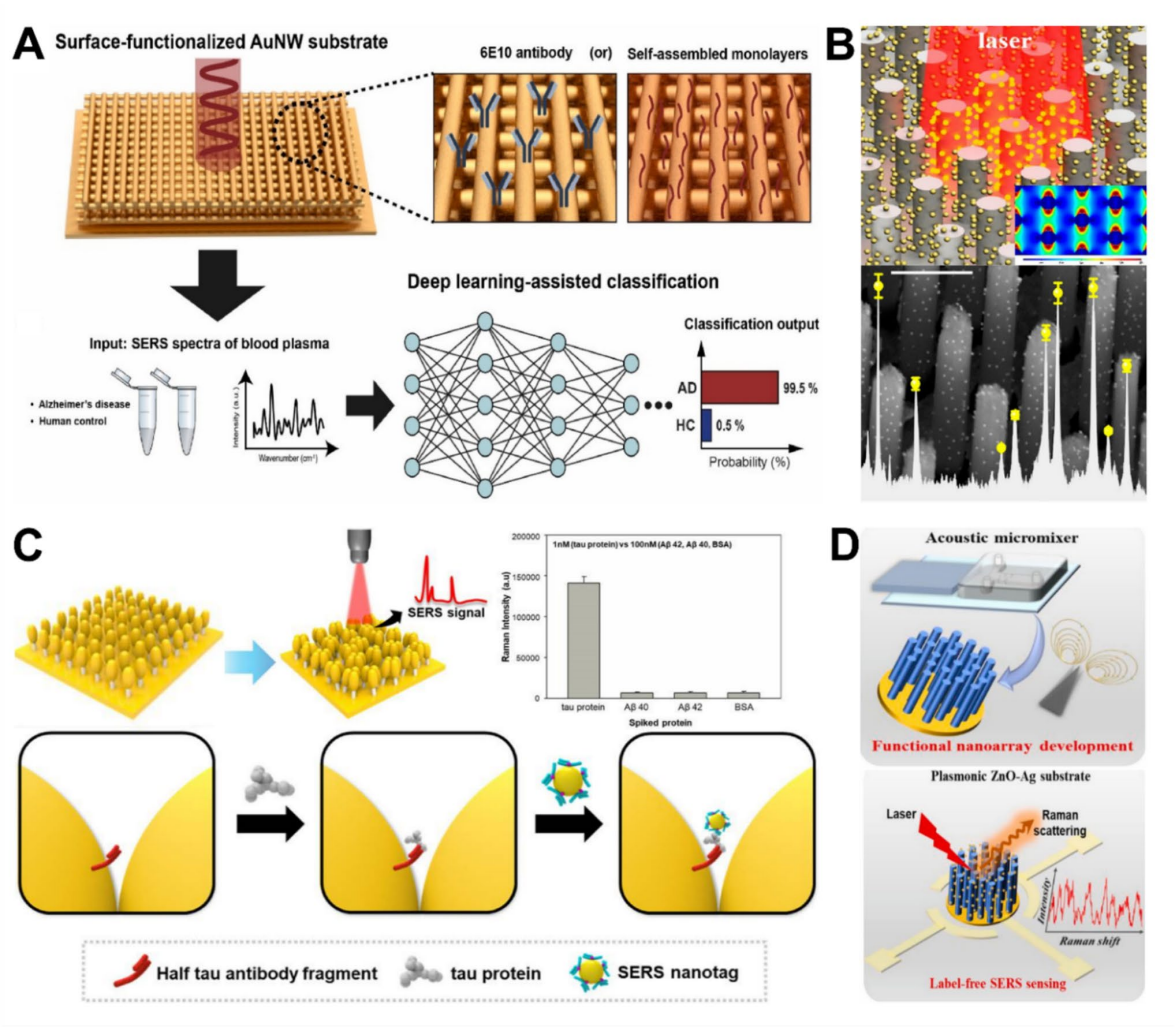
Illustration of nanopatterned SERS detection platforms for AD biomarkers detection. **A** Deep learning-assisted SERS platform based on Au nanowires functionalized with antibodies for the detection of Aβ42 from AD and human control samples. Reprinted with permission from ref. (Kim et al. 2024). **B** Graphical representation and scanning electron microscopy image of the SERS platform based on periodical, hexagonal-packed AuNP-conjugated Silicon nanorods array for the detection of long Aβ fibrils at a single fibril level. Reprinted with permission from ref. (Lin et al. 2017). Copyright 2017 American Chemical Society. **C** Illustration of a SERS sensor based on plasmonic nanopillars for tau protein detection. Reprinted with permission from ref. (Yang et al. 2022). **D** Label-free SERS acoustofluidic and multimodal system for the dual detection of both Aβ and tau from AD patients and healthy controls. Reprinted with permission from ref. (Hao et al. 2022)

Taking advantage of the LSPR properties that AuNPs possess, Ly and Park (2020) detected Aβ42 at a concentration as low as 1 pg/ml from diluted CSF by employing SERS as an ultrasensitive detection method. The group developed a highly sensitive sensor based on AuNPs thin films deposited on a polyethylene terephthalate substrate. Prior to the thin film formation, AuNPs were functionalized with 11-mercaptoundecanoic acid and deposited on the PET substrate by the Langmuir–Blodgett technique. Highly specific monoclonal antibodies were immobilized on the metallic layers by the aid of streptavidin. By measuring the changes in plasmonic peak absorbance, the attachment of Aβ42 to the specific antibodies was demonstrated, at an optimal AuNPs diameter of 9 nm. An antibody-based LSPR nanosensor was also developed by Haes et al. (2004) to detect amyloid-derived diffusible ligands (ADDLs) and a binding constant of 3 × 10^7^ M^−1^ was measured. The nanoplatform comprised of polystyrene nanospheres of 390 nm diameter drop-coated onto glass slides and covered with a layer of Ag nanotriangles with a height of 28–29 nm and fabricated using nanosphere lithography. Using the same lithographic technique, Yu et al*.* (2020) fabricated a hybrid SERS platform based on graphene and Au nano-pyramids on a monolayer of polystyrene balls with a diameter of 200 nm deposited on silica (Si) substrate. A detection limit down to 10^–18^ M was determined and the co-location between Aβ and a hot spot was demonstrated. Taking advantage of the ordered assembling of polystyrene nanospheres on a Si substrate, a SERS sensor based on hexagonal-packed Si nanorod arrays in conjunction with AuNPs (presented in Fig. 3b) was fabricated by Lin et al*.* (2017)*.* The nanoplatform reached a detection limit of 0.13 μM for Aβ oligomers and 1.3 μM for Aβ fibrils.

The autopsy of AD patients revealed high concentrations of Zn^2+^ and Ca^2+^ ions together with Al^3+^ and Aβ in the senile plaques. Using a Au-based substrate with a SAM of 16-Mercaptohexadecanoic acid and combining SERS with AFM techniques, Xie et al*.* (2021) demonstrated that sub-stoichiometric metal cations (Zn^2+^, Ca^2+^, Al^3+^) presence can induce the aggregation of Aβ42, at a pH of 7.4. Eremina et al*.* (2024) took advantage of the aggregation role of Zn^2+^ and Cu^2+^ to identify and quantify Aβ aggregates using two types of SERS substrates: AgNP/chitosan films decorated with AgNPs, and AgNP-based substrates fabricated by laser induced deposition method. A robust quantification of Aβ aggregates with a limit of detection of 1.5 nM was reported in case of the AgNP/chitosan films, while the second SERS platform showed a detection down to 15 pM.

Another interesting class of detection platforms is based on the integration of aptamers in contactless, light reflectance spectroscopy based sensors, such as the one designed by Amouzadeh Tabrizi et al*.* (2019). The biosensors incorporated Au-coated nanoporous anodic alumina walls modified with (3-aminopropyl) trimethoxysilane (NAA-NH2) aptamers, and methylene blue as the photo-probe. Interferometric reflectance spectroscopy (IRS) was used as detection technique and an experimental LoD of 0.02 μg/mL of Aβ oligomers was found. The progression monitoring of AD stages using a SERS nanoplatform was achieved by Kim et al*.* (2024) by identifying the oligomerization steps of Aβ42 with an accuracy of 96–100% in plasma samples. The SERS substrate consisted of an antibody-functionalized Au-nanowires substrate fabricated by solvent-assisted nanotransfer printing technique. Deep learning-assisted classification was performed with outstanding accuracies, as shown in Fig. 3a.

A sensitivity of 2.76 μg (or 1.388 mg/ml) has been reached in the case of a fluorescence-based sensor incorporating Ag thin film and Zn nanoflowers with hexagonal tips in combination with Thioflavin T, a highly specific fluorophore for amyloid detection (Akhtar et al. 2017).

As microfluidic approaches, Chou et al*.* used a SERS nanofluidic trapping device to detect Aβ in different conformational states and to differentiate Aβ from other existing confound proteins (such as insulin and albumin) in CSF. The size-dependent trapping mechanism relies on the use of a nanochannel of 40 nm depth to localize, concentrate and aggregate AuNPs of 60 nm in diameter and to confine the target analyte (Chou et al. 2008; Benford, et al. 2008). A PDMS nanofluidic-based system was employed by Choi et al. to investigate Aβ aggregation and its evolution at concentrations ranging from 10 fM to 1 μM. The SERS device is based on AuNPs of 80 nm diameter immobilized onto an APTMS-modified glass slide bonded to the PDMS mold composed of micro/nanofluidic junctions (Choi et al. 2012). A new screening platform for the selective detection of Aβ42 in simulated CSF was recently reported by Jaiswal et al*.* and comprises of a purine-based ligand-AgNPs active SERS substrate, integrated on a PDMS film with microchannel and sealed with a flat PDMS cover. A sensitivity of 10^–15^ M was achieved and a LOD of 10^–13^ M (Jaiswal et al. 2024).

#### Tau proteins

Various types of rationally designed biosensors (optical, electrochemical and piezoelectric) have demonstrated a LoD down to fM concentration to screen tau protein in serum, artificial CSF (aCSF) or plasma, and have been summarized by Ameri et al*.* (2020). Among these, the optical biosensors heavily rely on the integration of a biorecognition element, such as monoclonal anti-tau antibody (tau-mAb).

A LoD of 10 pg/mL for commercially available tau protein was reported by Vestergaard et al*.* (2008) when using a multi-spot LSPR-based immunochip with amino-modified silica NPs with upper and lower layers of Au of 40 nm and 30 nm in thickness, respectively. 4,4-Dithiodibutyric acid (DDA) was used as a linker for the immobilization of tau-mAb. The reported LoD suggested that the designed LSPR chip has a high specificity and sensitivity, lower than the cut-off value of 195 pg/mL of tau in CSF clinical samples.

A SPR biosensor with a LoD of 125 pM has been reported by Lisi et al*.* (2017). Although the LoD of SPR sensors is limited to nanomolar range, an incorporation of Multi Walled Carbon Nanotubes (MWCNTs) decorated with secondary monoclonal antibody (mAb_2_) lead to a decrease in the LoD of human tau protein from aCSF and buffer solution to pM range, as requested in clinical protocols. Besides the incorporation of MWCNTs into the classic immuno-sandwich, another element that contributed to attain a pM LoD was the high availability binding of mAb_2_. This approach could pave the road to a further improvement in the analytical performances of SPR-based biosensors for tau protein detection.

In comparison to Lisi et al. (2017), Kim et al*.* (2016) detected human tau381 isoform in undiluted plasma with a LoD of 10 fM by using a new DNA aptamer/antibody sandwich assay. The SPR Au-based chip was functionalized with a mixed monolayer of 11-mercaptoundecanoic acid (MUA) and 11-mercaptoundecanol (MUD) on which the tau specific 5’-amine modified DNA aptamer covalently bonded. The tau proteins from buffer and plasma were bound by the aptamers, followed by the subsequent binding of anti-tau antibodies. A lower LoD of tau381 (2.2 fM) was recently reported by Dang et al*.* (2023) when using a SERS nanoplatform based on DNA aptamers labelled with Raman reporters. The plasmonic substrate incorporated uniform AuNPs with an average size of 64 nm in the form of Au nanopopcorns fabricated *via* thermal evaporation.

Another SERS sensor designed for the detection of tau protein in blood plasma of AD patients was reported by Yang et al*.* (2022). The diagnostic platform incorporated plasmonic head-flocked nanopillar arrays fabricated on silicon substrates using a maskless reactive ion etching (RIE) process and oxygen plasma treatment. The nanostructures were covered with Au film by electron beam evaporation technique, while another Au layer was deposited on top of it. The Au nanostructures were functionalized with half antibody fragments, followed by the binding of SERS nanotags on 15 nm AuNPs. The high specificity and selectivity of the SERS sensor was detected on a spiked sample containing tau protein with 1 nM concentration, while the others proteins (Aβ40, Aβ40 and bovine serum albumin) were present in a concentration of 100 nM, as shown in Fig. 3c. This reduced binding distance yielded a significant improvement compared to using whole antibodies due to a more efficient plasmonic coupling between the nanopillar heads and SERS nanotags. In AD patients, a LoD of 951 fM was reached. This low LoD made it possible to distinguish all AD patients from healthy controls.

An innovative immunoassay SPR-based approach involved the use of a Au/Cr-coated multimode optical fibers with immobilized antibodies for T-tau and P-tau detection in human sera (Vu Nu et al. 2018). The approach involved the replacement of plastic cladding of the fiber with 1 nm Cr/40 nm Au coating deposited using evaporation technique. The anti-tau antibodies were immobilized on the carboxyl-activated surface of the SPR fiber sensor and selectively captured tau441 and P-tau199/202 in various concentrations. A LoD of 2.4 pg/mL (0.53 fM) for T-tau and 1.6 pg/mL (1.3 pM) for P-tau were attained during calibration protocol, and values of 61.91 ± 42.19 ng/mL and 50.25 ± 18.17 ng/mL respectively were obtained in samples from AD patients. The calibrated sensor yielded an average concentration of T-tau and P-tau sixfold higher and threefold higher than in controls, when human sera from the blood samples of 40 human subjects with age over 65 were tested. The low sensitivity and user-friendly design could be the key for on-demand applications, and early-stage diagnosis of AD could greatly benefit from this type of immunoassay SPR sensor.

A first attempt to directly identify P-tau using label-free SERS and no antibodies was reported by Ma et al*.* (2021). Their work focused on the SERS fingerprint of *in situ* amino acid phosphorylation and discrimination between tau410 with phosphorylated site at Serine 214 (P-tau214) and 396 (P-tau396) using SERS coupled with molecular dynamics simulations. In comparison with previous reports, this group used an Ag/glass-based platform with a self-assembled layer of iminodiacetic acid (IDA) spacer which ensured a soft immobilization of P-tau on the Ag surface. In comparison to these results, Zhang et al*.* (2023) managed to detect the P-tau at Serine 396 (P-tau396) and 404 (P-tau404) in whole blood samples by using a SERS dual-mode magnetic sensor with a LoD of 1.5 pg/mL. The dual detection platform relies on superparamagnetic iron oxide NPs (SIONPs) modified with antibodies selective for P-tau396 and P-tau404. Horseradish peroxidase (HRP)-labeled antibodies and AuNPs functionalized with 4-MPA linker were used as colorimetric and Raman dual output.

#### Multiplexed AD biomarkers

Nanostructured platform-based multiplexed detection of Aβ and tau has been better explored compared to colloidal approaches. Taking into consideration the multiple advantages of solid SERS substrates for protein research, such as the lack of denaturation, several groups fabricated solid-based SERS substrates for multiplex detection of AD biomarkers to increase the informative value through the use of biomarker ratios, as discussed below.

In their work, Park et al. (2020) designed carboxylic-acid-functionalized and graphitic nanolayer coated 3D SERS substrates (CGSSs) with 2 or 4 Au nanowire array sheets using solvent-assisted transfer printing (S-nTP) technique and PDMS as a transfer medium. The master mould used in the fabrication process contained trenches with a width of 1 μm and sidewall widths of 0.25 μm. Inside every trench, block polymers stripes with a diameter of 15 nm and gap of 20 nm were fabricated by direct self-assembled method. Every new nanowire sheet was stacked perpendicular on the previous one. By employing a perpendicular laser irradiation, huge local E-fields were generated with good reproducibility. Aβ and tau markers were uniformly immobilized onto a carboxylic-acid-functionalized layer. An EF of 5.5 × 10^5^ was achieved and a very small spot-to-spot signal variation was determined, down to ≈ 6%. Taking into consideration the intensity of phenyl marker band at 1002 cm^−1^ and the amide S band at 1380 cm^−1^, a qualitative analysis of tau protein and Aβ was made.

Hao et al*.* developed an integrated multimodal sensor using acousto-fluidics to isolate and detect Aβ peptides and tau protein (Fig. 3d). The new diagnostic system (ADx) combines the advantages of SERS and electrochemical sensors and is based on ZnO nanoarray-patterned Au electrodes, in a more accurate, stable and reproducible system. The authors examined the differences occurred in blood plasma samples of 10 AD patients and 7 healthy controls (Hao et al. 2022).

The innovative combination between SERS and convolutional neural networks (CNNs) was employed by Yu et al*.* (2022) to detect AD biomarkers and to analyze the biochemical changes in human CSF. The SERS measurements were done on 20 AD CSF samples and 10 healthy patients, employing an enhancement substrate with periodic and uniform Au nanopyramid structure with a diameter of 200 nm, fabricated using sphere lithography. Using this joint approach, an accuracy of 92% in disease diagnosis was achieved. A more recent report of Wu et al*.* (2024) discussed the impact of the Au nanopyramid size and gap spacing on the EF and detection limits for tau, P-tau proteins and Aβ42 polypeptides. The label-free SERS platform based on graphene-Au nanopyramids was fabricated by colloidal lithography and several micro/nanofabrication techniques, such as e-beam and magnetron sputtering deposition methods, lift-off and etching. PS microspheres with diameters of 500, 350, 500, and 200 nm, were respectively used. The FDTD simulations demonstrated that a larger pyramid size and smaller gap distance facilitated a higher SERS EF. LoD for T-tau and P-tau reached 10^−15^ M, while in case of the Aβ42 it was 10^−14^ M.

A more recent study which combines SERS and machine-learning algorithms reported the detection of Aβ40, Aβ42, P-tau, and T-tau biomarkers from blood plasma by using an innovative SERS immunoassay platform (Resmi et al. 2024). The work proposes an antibody-immobilized aluminium SERS substrate based on AuNPs which spans a wide detection range from aM to μM concentration, able to clearly differentiate mild cognitive impairment from AD and healthy controls.

The use of neural networks and machine learning in the analysis of SERS data is still in its infancy, as can be seen from the low number of references, but it is increasingly integrated into nano-spectroscopic platforms for biomarker detection. AI approaches are primarily applied to analyze high-dimensional spectral datasets enabling automated feature extraction, noise reduction, and reducing user-dependent bias in classification between control and AD samples. They also facilitate multiplexed biomarker detection by deconvoluting overlapping spectral features, thus supporting more complex diagnostic signatures rather than reliance on single-analyte peak intensity measurements.

## Potential of nano-based assays for clinical translation and AD diagnosis

Although solid-state and micro/nano-fabricated detection platforms overcome some limitations of colloidal assays by providing higher reproducibility and scalability, translation into clinically approved diagnostic tools requires more than analytical performance. Regulatory approval pathways demand demonstrable robustness, inter-batch consistency, scalable manufacturing, standardized quality control metrics, and validation in large, statistically powered clinical cohorts. In this context, reproducibility and manufacturability, rather than absolute detection limits, emerge as the primary bottlenecks for technological transfer.

Three-dimensional SERS substrates, graphene-based hybrids, and lithographically patterned nanopillars consistently achieve ultra-low detection limits. Yet, their fabrication often requires cleanroom facilities and multi-step protocols, raising cost and accessibility concerns. More importantly, the critical obstacles to clinical implementation lie in reproducibility and manufacturability: small variations in nanostructure geometry, deposition conditions, or surface functionalization yield substantial differences in enhancement factors, undermining batch-to-batch consistency. Consequently, despite their analytical promise, these platforms must overcome substantial challenges in fabrication standardization and large-scale production before they can support reliable, clinically meaningful biomarker detection.

Another key factor for SERS signal maximization is the enhancement factor. It is highly dependent on the experimental conditions’ variations and therefore difficult to compare across studies, but it remains a reference for the SERS performance. Consistent reference standards would help to overcome this issue, while a reliable parameter, namely the SERS performance factor (SPF) could represent a more reliable strategy in terms of objectivity and reproducibility of the SERS efficiency. Establishing standardized characterization protocols, analogous to quality control frameworks used in conventional immunoassays, will be essential for regulatory alignment.

Moreover, head-to-head comparisons across platforms are scarce, making it difficult to determine which design principles most effectively balance sensitivity, specificity, and clinical feasibility. To date, only a handful of studies have evaluated these devices with actual patient biofluids, and nearly none have included statistically significant cohorts. Many studies demonstrate performance primarily in buffered or spiked model systems, whereas validation in complex biological matrices such as cerebrospinal fluid (CSF), plasma, saliva, or urine is comparatively limited (Resmi et al. 2024; Georganopoulou et al. 2005; Dallari, et al. 2025; Milà-Alomà et al. 2019). In real biofluids, issues such as nonspecific adsorption, protein corona formation, matrix interference, and colloidal instability may significantly affect signal reproducibility and quantitative accuracy. Furthermore, batch-to-batch variability in nanoparticle/nanoplatform synthesis can strongly influence plasmonic enhancement and SERS response, raising concerns regarding inter-laboratory reproducibility and standardization (Mülhopt et al. 2018).

SERS- and SPR-based multiplex platforms demonstrated proof-of-concept feasibility; and impressive LoD values. However, in practice, multiplexing introduces substantial challenges due to the amplified chemical complexity: cross-reactivity between capture ligands, matrix interference, and spectral overlap that complicates data interpretation and often limits performance more than analytical sensitivity itself. Thus, increasing the number of detectable targets does not automatically translate into improved clinical discrimination unless selectivity and calibration are rigorously validated.

Although the reported ultra-low detection limits are often well below physiological concentrations of Aβ and tau species, typically present at pM–nM levels in CSF and low pg/mL levels in plasma, the clinical benefit of further sensitivity enhancement remains debatable (Tapia-Arellano et al. 2024; Lewczuk et al. 2018). In practice, diagnostic utility depends not only on analytical sensitivity but also on selectivity toward disease-relevant isoforms, robustness against biological variability, and the ability to discriminate pathological from physiological ranges in heterogeneous patient populations. In this context, the use of antibody- or aptamer-functionalized nanostructures supports selective capture in complex biofluids, addressing limitations related to nonspecific binding and signal instability, as a promising strategy for improving clinical relevance rather than merely lowering detection limits.

Practical considerations further influence translational potential. Material optimization, rational nanostructure design, and minimization of matrix-derived signal contributions remain important research directions. However, cost, instrumentation requirements, operational complexity, and compatibility with routine laboratory workflows are equally critical. The development of partially or fully automated sample pre-treatment protocols could reduce operator-dependent variability, enhance analytical reproducibility, and facilitate integration into standardized diagnostic pipelines.

Artificial intelligence (AI) and machine learning approaches are increasingly incorporated into SERS-based AD detection platforms (Li et al. 2025; Ashraf et al. 2025; Lee et al. 2026) to extract subtle spectral signatures and improve classification between healthy controls, MCI, and AD patients. From a translational perspective, AI offers clear advantages: it can compensate for substrate heterogeneity, reduce user-dependent spectral interpretation bias, and enable automated decision-making that may support decentralized testing. However, many reported models rely on limited training datasets, raising concerns regarding overfitting and limited generalizability across independent cohorts or fabrication batches. Reliable deployment requires standardized preprocessing protocols, transparent validation strategies, and multi-center clinical datasets. Furthermore, regulatory acceptance of AI-driven diagnostics depends on explainability, traceability, and rigorous clinical validation. Thus, while AI enhances analytical robustness and classification performance, its true clinical impact will depend on integration into standardized, reproducible analytical frameworks (Ajith et al. 2026) rather than on high accuracy metrics derived from small-scale experimental studies.

## Conclusions

This review highlights the promise of nano-based spectroscopic approaches in advancing the early detection of Alzheimer’s disease protein biomarkers. Traditional methods, such as cerebrospinal fluid sampling or neuroimaging, are effective but invasive, costly, and impractical for large-scale screening. Minimally invasive biofluids, including blood, saliva, and urine, offer superior accessibility and patient compliance, even though they contain much lower biomarker concentrations. Through plasmonic enhancement and engineered nanostructures, techniques such as SERS now enable detection of Aβ and tau species at concentrations well below their physiological ranges in CSF and peripheral biofluids. The adaptability of these platforms to multiplexing, label-free detection, and machine-learning-assisted analysis underscores their conceptual promise for minimally invasive, early-stage diagnostics.

However, the central limitation is no longer sensitivity. The primary obstacles to clinical implementation lie in reproducibility, standardization, and scalability. Variability in nanostructure fabrication, surface functionalization, and enhancement factor reporting undermines inter-laboratory comparability. Many systems remain validated only in buffered or spiked samples, with limited evaluation in complex biological matrices and small patient cohorts. Without standardized reference materials, harmonized performance metrics, and robust multi-center validation, impressive laboratory results cannot translate into reliable clinical diagnostics. In parallel, practical consideration, including cost, manufacturing under quality-controlled conditions, automation of sample preparation, and compatibility with routine laboratory workflows, must be addressed systematically.

The integration of artificial intelligence represents a powerful complementary strategy, capable of compensating for substrate heterogeneity and extracting subtle spectral signatures. Yet its clinical value depends on transparent validation frameworks, sufficiently large training datasets and regulatory compliance.

Future progress in nano-enabled AD diagnostics will therefore require a shift from proof-of-concept demonstrations toward reproducible device engineering, standardized analytical pipelines, and interdisciplinary collaboration between nanotechnologists, clinicians, data scientists, and regulatory experts. If these challenges are met, nano-spectroscopic biosensing could evolve from an experimental research domain into a clinically meaningful tool for early and minimally invasive Alzheimer’s disease detection.

## Data Availability

No datasets were generated or analysed during the current study.
